# Cholesterol 25‐Hydroxylase Deficiency Provides an Optimal and Durable Protective T Helper 1 Immunity via Interleukin‐12–Interleukin‐2 Axis in BCG‐Vaccinated Mice

**DOI:** 10.1002/mco2.70989

**Published:** 2026-09-30

**Authors:** Hagyu Kim, Eunsol Choi, Kee Woong Kwon, Hong‐Hee Choi, Kyungmin Kim, WeonSeok Jung, Sung Jae Shin

**Affiliations:** ^1^ Department of Microbiology, Institute For Immunology and Immunological Disease, Graduate School of Medical Science, Brain Korea 21 Project Yonsei University College of Medicine Seoul South Korea; ^2^ Department of Microbiology Yonsei University College of Medicine Seoul South Korea; ^3^ Department of Microbiology, College of Medicine Gyeongsang National University Jinju South Korea; ^4^ Department of Microbiology, Graduate School of Medicine Yonsei University College of Medicine Seoul South Korea; ^5^ Department of Biomedical Sciences Yonsei University College of Medicine Seoul Republic of Korea

**Keywords:** Bacillus Calmette‐Guérin, cholesterol 25‐hydroxylase, dendritic cell, *Mycobacterium tuberculosis*, T helper 1 response, tuberculosis

## Abstract

Bacillus Calmette‐Guérin (BCG) remains the only licensed vaccine for tuberculosis (TB), yet its efficacy wanes over time due to insufficient durability of vaccine‐induced immunity. However, mechanisms constraining BCG vaccine‐induced dendritic cell (DC)‐mediated T cell priming are poorly understood. Cholesterol 25‐hydroxylase (CH25H) converts cholesterol into 25‐hydroxycholesterol (25‐HC) that modulates antigen‐presenting cell imprinting and innate immune responses. Here, we investigated whether CH25H regulates DC function and vaccine‐induced protective immunity against *Mycobacterium tuberculosis* (Mtb). We found that CH25H deficiency enhanced DC activation, characterized by increased expression of the costimulatory molecules CD80 and CD86, elevated interleukin (IL)‐12p70 production, and reduced IL‐10 secretion, resulting in augmented T cell proliferation and robust Th1 cytokine responses. Transcriptomic analyses of CH25H‐deficient and 25‐HC‐treated DCs showed that an SREBP2‐associated program may contribute to *Il12a* expression. BCG vaccination conferred enhanced protection in *Ch25h^−/−^* mice, including reduced Mtb burden and pulmonary inflammation. BCG‐vaccinated *Ch25h^−/−^* mice developed more durable protective immunity and increased frequencies of CD103^+^CD69^+^ tissue‐resident memory T cells, along with sustained IL‐2‐producing T cell responses. Collectively, these findings identify CH25H as a negative regulator of DC‐mediated Th1 immunity and suggest that targeting CH25H represents a potential host‐directed immunomodulatory strategy to enhance the long‐term protective immunity of TB vaccination.

## Graphical abstract

CH25H deficiency permits optimal dendritic cell activation following BCG exposure, characterized by increased IL‐12p70 production. In vivo, CH25H deficiency promotes enhanced BCG‐mediated protection and sustained IL‐2 producing T cell response.

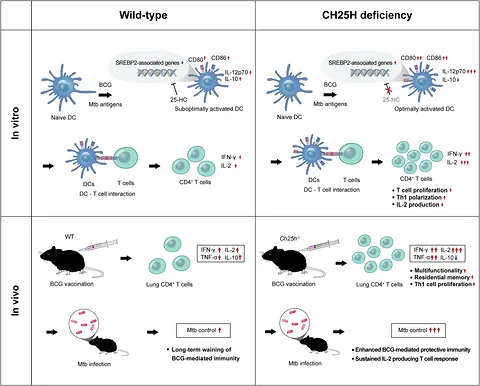

## 1 Introduction

Despite global efforts, tuberculosis (TB) remains a leading infectious killer worldwide [1]. Bacillus Calmette‐Guérin (BCG) vaccination is the only available licensed vaccine against TB [2]. However, after more than a century of clinical use, BCG remains suboptimal in conferring durable protection against TB [3, 4]. While BCG effectively prevents severe childhood TB, its efficacy against adult pulmonary TB varies greatly among populations and wanes over time [5]. This heterogeneity and short‐lived immunity have been attributed to environmental mycobacterial exposure, strain variability, and host immune diversity [6, 7]. Furthermore, although BCG induces robust T helper 1 (Th1) responses that are critical for TB control, it is insufficient to establish durable lung tissue‐resident memory T cells (TRMs) essential for protective immunity [8]. In addition, the limited antigenic repertoire of BCG only partially overlaps with that of virulent *Mycobacterium tuberculosis* (Mtb), resulting in insufficient induction of multifunctional T cells required for sustained protection [9, 10]. These limitations highlight the urgent need for next‐generation vaccine strategies that can elicit durable, tissue‐specific, and functionally diverse immune responses that surpass those elicited by conventional BCG vaccination [11].

Cholesterol and its oxidized derivatives, collectively known as oxysterols, play pivotal roles in immune regulation, including inflammation, immune cell differentiation, and migration [12, 13]. Among multiple derivatives, 25‐hydroxycholesterol (25‐HC) is produced by the enzyme cholesterol 25‐hydroxylase (CH25H), which is primarily expressed in innate immune cells and localized to the endoplasmic reticulum [14]. CH25H‐mediated production of 25‐HC plays a pivotal role in suppressing viral membrane fusion and DNA replication in various viral infection models, including Ebola virus and SARS‐CoV‐2 [15, 16]. Multiple studies demonstrated that the expression of CH25H is dependent on Type I interferon (IFN) signaling and the JAK/STAT1 pathway, which are essential factors for initiating antiviral immune responses [17].

Furthermore, over the past decade, studies have revealed that CH25H and 25‐HC can influence immune responses against bacterial infections. In macrophages, *Listeria monocytogenes* infection induced CH25H expression in a Type I IFN‐dependent manner, promoting pathogen survival [18]. In a murine model of *Streptococcus pneumoniae* infection, CH25H‐deficient mice exhibited enhanced phagocytic activity and bacterial clearance, resulting in attenuated lung pathology compared with wild‐type (WT) mice [19]. CH25H has been suggested to suppress interleukin (IL)‐1β expression and promote the development of immunosuppressive macrophages [20], and mechanistically, it modulates inflammasome activity and IL‐1 signaling downstream of Type I IFN, acting as a negative regulator of inflammatory responses [20, 21]. In contrast, exogenous 25‐HC can activate the NLRP3 inflammasome [22], enhance IL‐1β production in microglia [23], and amplify inflammatory signaling by promoting transcriptional activation of proinflammatory genes [24, 25]. In addition, 25‐HC conferred innate resistance to bacterial infection through modulation of cell surface accessible cholesterol [26]. Furthermore, CH25H can influence host immune responses during mycobacterial infection. CH25H and 25‐HC were upregulated in Mtb‐infected macrophages and lungs in mice [27]. CH25H‐derived oxysterols mediate eosinophil recruitment to the lungs via GPR183 during Mtb infection [28]. Additionally, CH25H expression was upregulated during pulmonary *Mycobacterium marinum* infection in mice, resulting in the formation of foam cells and aggravated lung pathology [29]. However, the impact of CH25H on host immune responses during Mtb infection remains poorly understood.

Beyond its role in innate immune regulation, accumulating evidence suggests that CH25H also critically shapes adaptive immune responses. IL‐27‐induced CH25H functions as a metabolic checkpoint that limits T cell expansion via 25‐HC [30]. Additionally, 25‐HC suppressed SIV antigen‐specific CD4^+^ lymphocytes, impairing IL‐2 and TNF‐α production [31]. Loss of CH25H reduces 25‐HC in the tumor microenvironment, weakening immunosuppressive macrophages and enhancing T cell responses [32]. These findings indicate that CH25H can suppress adaptive immune activation. Given the essential role of CD4^+^ T cells in protective anti‐TB immunity [33], the influence of CH25H on CD4^+^ T cell responses is likely to be an important factor in host defense against Mtb. *Ch25h* ^−/−^ mice exhibit altered immune characteristics, including enhanced IL‐1 responses and improved sterol regulatory element‐binding protein 2 (SREBP2)‐dependent IgA production [23, 34], implying that CH25H may disrupt the optimal induction of Mtb‐specific adaptive immune responses. As dendritic cells (DCs) are pivotal regulators of anti‐TB immunity, orchestrating host defenses in both infection and vaccine‐induced protection models [35, 36], investigating the role of CH25H in DCs could further advance understanding of protective immune response of BCG. Taken together, since CH25H is rapidly induced in DCs upon innate stimulation and can modulate antigen‐processing pathways, we hypothesized that CH25H acts as a cell‐intrinsic checkpoint that limits BCG‐driven DC priming and long‐term protective T cell immunity.

Here, we showed that CH25H limits optimal host protective immunity to Mtb by suppressing the T cell‐stimulatory capacity of DCs. *Ch25h^−/−^* bone marrow‐derived DCs (BMDCs) displayed increased expression of costimulatory molecules CD80 and CD86 and elevated IL‐12p70 production. Although *Ch25h^−/−^* mice did not exhibit intrinsic resistance to Mtb infection, BCG vaccination elicited more durable Mtb‐specific Th1 responses, characterized by sustained IL‐2 production. Consequently, BCG‐vaccinated *Ch25h^−/−^* mice exhibited significantly reduced pulmonary Mtb loads and inflammation compared with WT controls. Collectively, our findings suggest CH25H as a negative immune regulator of BCG‐induced protective immunity and highlight the CH25H/25‐HC axis as a potential pathway associated with durable vaccine‐induced protection against TB.

## 2 Results

### 2.1 CH25H Deficiency Enhances DC Activation and Th1‐Polarizing Cytokine Responses to Mycobacterial Antigens

CH25H is an IFN‐responsive metabolic enzyme that is rapidly induced in DCs upon TLR stimulation, suggesting that CH25H is embedded in early DC activation [17]. Moreover, CH25H was linked to DC‐T cell interaction in vivo, as CH25H in DCs can reshape antigen processing and regulate T cell activation, suggesting the potential for CH25H to tune DC functional outputs relevant to adaptive immunity [37]. To determine whether CH25H deficiency alters DC activation and cytokine responses to mycobacterial antigens, BMDCs were generated from WT and *Ch25h^−/−^* mice using granulocyte–macrophage colony‐stimulating factor (GM‐CSF) for 8 days and subsequently stimulated with lipopolysaccharide (LPS), purified protein derivative (PPD), antigen 85B (Ag85B), or BCG. Then surface marker expression and cytokine production were assessed (Figure 1A). Harvested BMDCs were analyzed according to the gating strategies (Figure S1A). CH25H deficiency did not affect BMDC differentiation, as comparable BMDC purity was observed between WT and *Ch25h^−/−^* BMDCs (Figure 1B). Notably, *Ch25h^−/−^* BMDCs showed elevated expression of costimulatory molecules CD80 and CD86 compared with WT BMDCs following stimulation with antigens and BCG exposure (Figure 1C). In contrast, the expression levels of MHC‐I and MHC‐II were not significantly upregulated in *Ch25h^−/−^* BMDCs following stimulation (Figure S1B). Additionally, stimulated *Ch25h^−/−^* BMDCs exhibited increased production of IL‐12p70, IL‐1α, and IL‐1β, along with reduced production of IL‐10, suggesting a cytokine milieu favoring Th1 differentiation [38] (Figure 1D). The secretion of TNF‐α, IL‐6, IL‐23, and TGF‐β was not significantly altered in stimulated *Ch25h^−/−^* BMDCs (Figure S1C). To investigate whether these cytokine changes were DC‐specific, we next examined cytokine production in bone marrow‐derived macrophages (BMDMs) following mycobacterial antigen stimulation. In line with a previous report, *Ch25h^−/−^* BMDMs produced higher IL‐1β than WT BMDMs [20], but showed no difference in IL‐10 and failed to produce IL‐12p70 with stimulation (Figure S2A). Consistent with previous reports [39, 40], both WT and *Ch25h^−/−^* BMDMs produced undetectable levels of IL‐12p70, with no significant difference in IL‐10 production between WT and *Ch25h* ^−/−^ BMDMs following either BCG exposure or Mtb infection (Figure S2B). Therefore, we hypothesized that DCs, rather than macrophages, are the primary immune cells responsible for modulating Th1 immune responses in *Ch25h^−/−^* mice. We next assessed temporal CH25H expression in DCs. CH25H expression peaked at 4 h after BCG exposure and declined thereafter (Figure 1E). In addition, IL‐12p70 production in *Ch25h^−/−^* BMDCs increased during the early phase of BCG exposure, whereas IL‐10 levels declined between 8 and 24 h post‐BCG exposure (Figure 1F). These findings suggest that early CH25H expression in BCG‐exposed BMDCs may contribute to the subsequent suppression of IL‐12p70 and induction of IL‐10, reflecting their reciprocal regulation [41, 42]. To further investigate the immunoregulatory role of CH25H, we treated BMDCs with exogenous 25‐HC, an oxysterol generated by CH25H from cholesterol. Exogenous 25‐HC significantly reduced IL‐12p70 production in both WT and *Ch25h^−/−^* BMDCs, indicating that 25‐HC functions as a downstream effector of CH25H capable of suppressing immune responses in BMDCs (Figure 1G). Collectively, these results suggest that CH25H influences DC responses to mycobacterial antigen stimulation, implying their potentiation to limit optimal Mtb‐specific Th1 cell differentiation.

**FIGURE 1:**
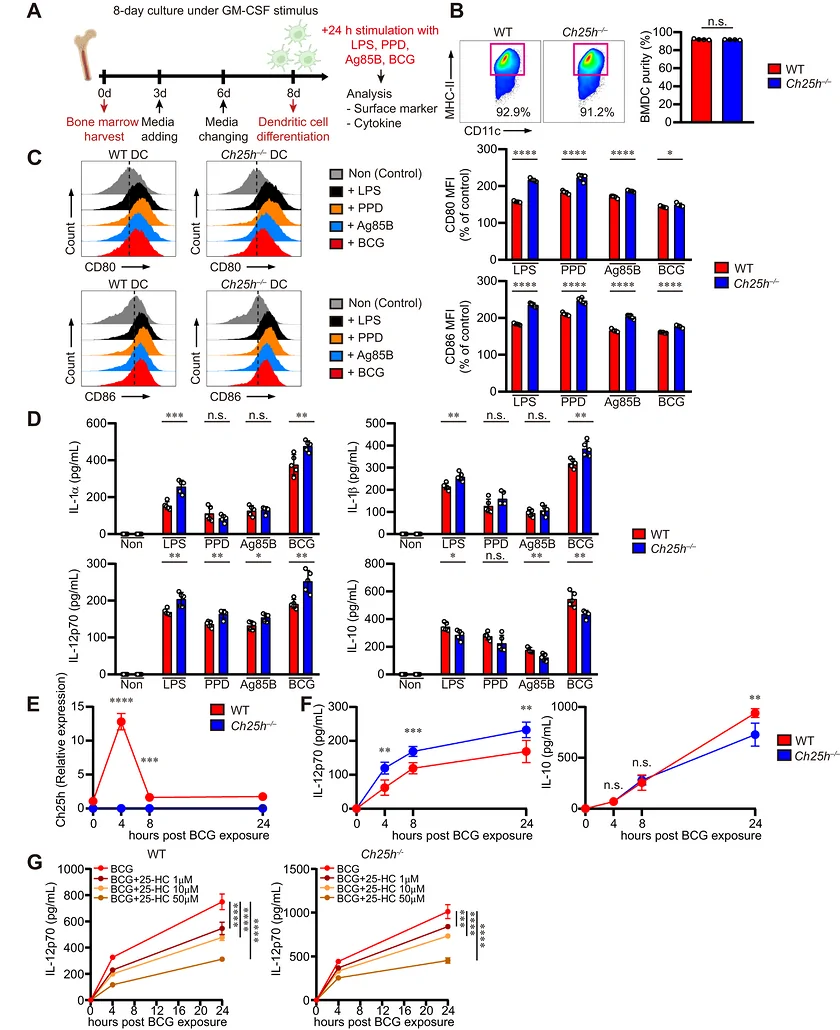
Comparative expression profiles of costimulatory molecules and cytokine production in BMDCs from *Ch25h* ^−/−^ and WT mice in response to BCG and mycobacterial antigens. (A) Experimental design for in vitro analysis of BMDC differentiation with WT and *Ch25h* ^−/−^ mice. (B) The purity of harvested CD11c^+^MHC‐II^+^ BMDCs. (C) Relative mean fluorescence intensity (MFI) values (% of unstimulated controls) of CD80 and CD86 in BMDCs. (D) The expression levels of IL‐1α, IL‐1β, IL‐12p70, and IL‐10 in supernatants of stimulated BMDCs. (E) Expression levels of CH25H gene in WT and *Ch25h* ^−/−^ BMDCs following BCG exposure at 0, 4, 8, and 24 h postinfection. (F) Expression levels of IL‐12p70 and IL‐10 cytokines in WT and *Ch25h* ^−/−^ BMDCs following BCG exposure at 0, 4, 8, and 24 h postinfection. (G) The expression levels of IL‐12p70 in WT and *Ch25h* ^−/−^ BMDCs with exogenous 25‐HC treatment. Data are presented as the mean ± SD of four or five biological replicates per group. (B–F) Data were analyzed unpaired *t*‐test. (G) Data were analyzed by one‐way ANOVA with posthoc Tukey's test. *n.s*., not significant; *p < 0.05, **p < 0.01, ***p < 0.001, ****p < 0.0001.

To investigate the downstream mechanism underlying the elevated IL‐12p70 production in *Ch25h* ^−/−^ BMDCs, we performed mRNA sequencing of WT and *Ch25h* ^−/−^ BMDCs after BCG exposure, as well as *Ch25h* ^−/−^ BMDCs treated with exogenous 25‐HC (Figure S3A). Comparative heat map analysis identified overlapping genes reciprocally regulated by CH25H deficiency and 25‐HC treatment (Figure S3B). Reactome pathway analysis showed that overlapping genes upregulated by CH25H deficiency and suppressed by 25‐HC were most significantly enriched in cholesterol biosynthesis, whereas the oppositely regulated gene set showed no significant gene‐gene interaction network (Figure S3C). GSEA analysis supported enrichment of cholesterol homeostasis in *Ch25h* ^−/−^ BMDCs following BCG exposure (Figure S3D). Consistent with the role of SREBP2 as a key regulator of cholesterol biosynthesis [43], SREBP2‐associated genes were elevated in BCG‐exposed *Ch25h* ^−/−^ BMDCs and attenuated by exogenous 25‐HC. Notably, *Il12a* showed a similar pattern, with higher expression in *Ch25h* ^−/−^ BMDCs and reduction following 25‐HC treatment (Figure S3E–J). These data suggest that loss of CH25H may enhance an SREBP2‐related transcriptional program, potentially contributing to increased *Il12a*/IL‐12p70 production.

### 2.2 CH25H Deficiency in DCs Promotes Rapid Proliferation and Th1 Polarization of CD4^+^ T Cells

To confirm whether *Ch25h^−/−^* DCs can polarize T cells toward the Th1 phenotype and CH25H deficiency can alter T cell responses independently from DCs, we conducted coculture experiments using DCs and T cells from allogeneic mice. BMDCs and splenic T cells were obtained from C57BL/6J, *Ch25h^−/−^*, and BALB/c mice. In the first experiment, C57BL/6J and *Ch25h^−/−^* BMDCs were cocultured with BALB/c splenic T cells to initiate allogeneic T cell responses. In the second experiment, C57BL/6J and *Ch25h^−/−^* splenic T cells were cocultured with BALB/c BMDCs. DCs were stimulated with PPD for 24 h before coculturing with allogeneic T cells, which were stained with carboxyfluorescein succinimidyl ester (CFSE). After 3 days of coculture, cells and supernatants were harvested to investigate T cell proliferation and cytokine production (Figure 2A). Cocultured T cells were gated and analyzed according to the gating strategies attached (Figure S4). Compared with C57BL/6J DCs, *Ch25h^−/−^* DCs induced enhanced spontaneous proliferation of BALB/c T cells. CD4^+^ and CD8^+^ T cells incubated with *Ch25h^−/−^* DCs exhibited greater proliferation than C57BL/6J DCs (Figure 2B). Furthermore, BALB/c T cells incubated with *Ch25h^−/−^* DCs produced more IFN‐γ, TNF‐α, and IL‐2 after coculture, while IL‐10 production was not significantly different from T cells incubated with C57BL/6J DCs (Figure 2C). In contrast, no significant differences in proliferation were observed between C57BL/6J and *Ch25h^−/−^* T cells incubated with BALB/c DCs (Figure 2D). Likewise, *Ch25h^−/−^* T cells did not show elevated Th1 cytokine expression compared with C57BL/6J T cells (Figure 2E). We also found that unstimulated *Ch25h^−/−^* DCs failed to induce T cell proliferation or Th1 cytokine production, indicating that antigenic stimulation is required to initiate DC‐mediated T cell activation under CH25H‐deficient conditions (Figure S5). In contrast, BCG‐exposed *Ch25h^−/−^* BMDCs efficiently induced rapid proliferation of BALB/c T cells and robust Th1 cytokine production (Figure S6). Collectively, these results indicate that CH25H deficiency in DCs, rather than in T cells themselves, is responsible for the accelerated Th1 polarization of naive T cells.

**FIGURE 2:**
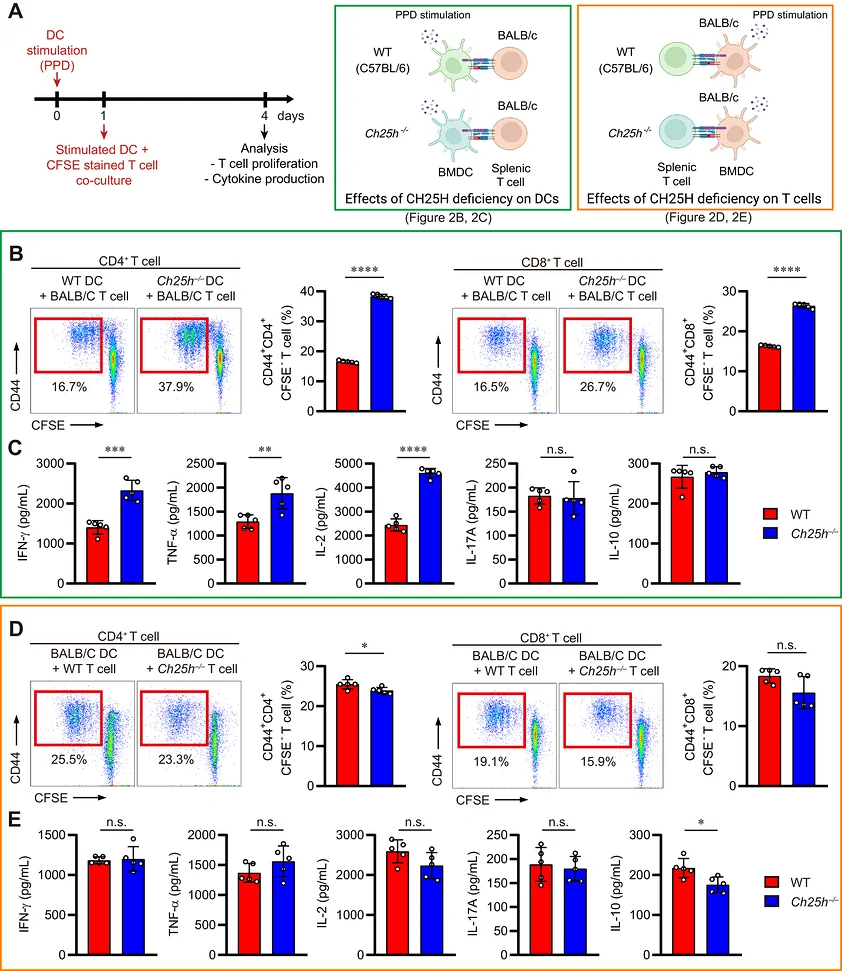
T cell proliferation and cytokine production status of splenic T cells cocultured with stimulated WT and *Ch25h* ^−/−^ BMDCs. (A) Experimental scheme for DC‐T cell coculture assay. WT and *Ch25h* ^−/−^ BMDCs were stimulated with Ag85B and cocultured with CFSE‐stained splenic T cells. After 3 days of coculture, flow cytometry analysis and ELISA were performed. (B) Proliferation of CD4^+^ and CD8^+^ T cells from BALB/c spleens cocultured with WT or *Ch25h* ^−/−^ BMDCs. (C) The expression levels of IFN‐γ, TNF‐α, IL‐2, IL‐17A, and IL‐10 in supernatants of BALB/c splenic T cells incubated with WT or *Ch25h* ^−/−^ BMDCs. (D) Proliferation of CD4^+^ and CD8^+^ T cell from WT or *Ch25h* ^−/−^ coculture with BALB/c BMDCs. (E) The expression levels of IFN‐γ, TNF‐α, IL‐2, IL‐17A, and IL‐10 in supernatants of BALB/c BMDCs incubated with WT splenic T cells or *Ch25h* ^−/−^ splenic T cells. The data are presented as the mean ± SD of five biological replicates per group. The significance of differences was determined, using unpaired *t*‐test. *n.s*., not significant. *p < 0.05, **p < 0.01, ***p < 0.001, ****p < 0.0001.

### 2.3 CH25H‐Deficient DCs Drive Robust Proliferation and Th1 Differentiation of Mtb‐Specific CD4^+^ T Cells

Since we found that CH25H deficiency in DCs enhances Th1 polarization and T cell proliferation through allogeneic DC‐T cell coculture assays, we next investigated whether CH25H‐deficient DCs could activate Mtb‐specific T cells. To evaluate this, we designed two independent DC‐T cell coculture models. In the first model, we utilized P25 TCR‐transgenic (Tg) mice, which contain CD4^+^ T cells expressing a Tg T cell receptor specific for peptide 25 (aa 240–254) of Mtb antigen Ag85B [36, 44]. Splenic T cells isolated from P25 TCR‐Tg mice were cocultured with WT (C57BL/6J) and *Ch25h^−/−^* BMDCs activated with the Ag85B peptide. In the second model, we harvested splenic T cells from BCG‐vaccinated WT mice 10 weeks postvaccination. BCG‐challenged splenic T cells were cocultured with WT and *Ch25h^−/−^* BMDCs stimulated with PPD. Splenic T cells were stained with CFSE and cocultured with the stimulated DCs for 3 days. Cells and supernatants were harvested to assess T cell proliferation and cytokine production (Figure 3A). We found that *Ch25h^−/−^* DCs induced spontaneous proliferation of P25 CD4^+^ T cells (Figure 3B) and upregulated the expression of IFN‐γ, IL‐2, and IL‐17A while downregulating the expression of IL‐10 (Figure 3C). Similar results were observed when *Ch25h^−/−^* DCs were cocultured with splenic T cells from BCG‐vaccinated mice. Specifically, *Ch25h^−/−^* DCs significantly increased the proliferation of BCG‐vaccinated CD4^+^ T cells (Figure 3D), upregulated IFN‐γ, IL‐2, and IL‐17A expression, and downregulated IL‐10 expression (Figure 3E). To further investigate whether the 25‐HC treatment could modulate this T cell response, DCs were pretreated with exogenous 25‐HC before Ag85B stimulation and subsequent coculture with P25 TCR‐Tg T cell. 25‐HC treatment reduced P25 CD4^+^ T cell proliferation (Figure S7A) and decreased IFN‐γ and IL‐2 expression (Figure S7B). Taken together, we demonstrated that CH25H‐deficient DCs are highly effective in promoting the proliferation and Th1 polarization of Mtb‐specific T cells.

**FIGURE 3:**
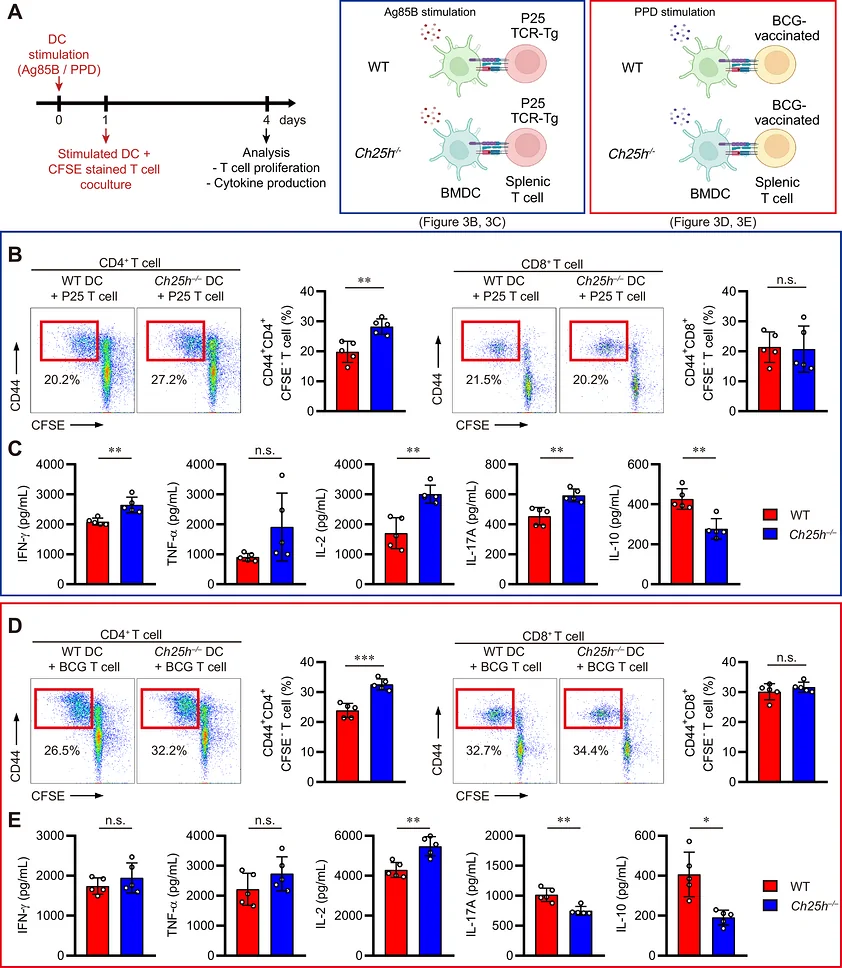
T cell proliferation and cytokine production status of Mtb‐specific splenic T cells cocultured with stimulated WT and *Ch25h* ^−/−^ BMDCs. (A) Experimental scheme for DC‐T cell coculture assay. WT and *Ch25h* ^−/−^ BMDCs were stimulated with Ag85B peptide or PPD and cocultured with CFSE‐stained splenic T cells from P25 TCR‐Tg mice or BCG‐vaccinated WT mice (sacrificed at 10 weeks postvaccination). After 3 days of coculture, flow cytometry analysis and ELISA were performed. (B) CD4^+^ and CD8^+^ T cell proliferation status in P25 TCR‐Tg splenic T cells incubated with WT or *Ch25h* ^−/−^ BMDCs. (C) The expression levels of IFN‐γ, TNF‐α, IL‐2, IL‐17A, and IL‐10 in supernatants of P25 TCR‐Tg splenic T cells incubated with WT or *Ch25h* ^−/−^ BMDCs. (D) CD4^+^ and CD8^+^ T cell proliferation status in BCG‐vaccinated WT splenic T cells incubated with WT or *Ch25h* ^−/−^ BMDCs. (E) The expression levels of IFN‐γ, TNF‐α, IL‐2, IL‐17A, and IL‐10 in supernatants of BCG‐vaccinated WT splenic T cells incubated with WT or *Ch25h* ^−/−^ BMDCs. The data are presented as the mean ± SD of five biological replicates per group. The significance of differences was determined using an unpaired *t*‐test. *n.s*., not significant. *p < 0.05, **p < 0.01, ***p < 0.001.

### 2.4 BCG Vaccination Enhances Antigen‐Specific T Cell Responses in *Ch25h^−/−^* Mice Compared With WT Mice

Based on our findings, we next explored whether the immunological changes induced by CH25H deficiency could enhance anti‐TB immune responses during Mtb infection. At 4 weeks postinfection, Mtb burdens and pulmonary inflammatory lesions were not significantly altered in Mtb‐infected *Ch25h^−/−^* mice (Figure S8A–C). While the numbers of pulmonary DCs and CD86 expression were upregulated in *Ch25h^−/−^* mice (Figure S8D), there was no significant difference in antigen‐specific immune responses against mycobacterial antigens PPD and Ag85B. Neither the total number of effector T cells (Figure S8E), the proportions of multifunctional IFN‐γ^+^ TNF‐α^+^, IFN‐γ^+^ IL‐2^+^, IFN‐γ^+^ TNF‐α^+^ IL‐2^+^ pulmonary CD4^+^ T cells (Figure S8F), the numbers of IFN‐γ^+^, TNF‐α^+^, and IL‐2^+^ CD4^+^ T cells (Figure S8G), nor the expression of cytokines including IFN‐γ, TNF‐α, IL‐2, and IL‐10 were significantly increased in the lungs of Mtb‐infected *Ch25h^−/−^* mice (Figure S8H). Despite the fact that CH25H is a well‐characterized IFN‐stimulated gene (ISG) and ISG expression has been linked to TB progression [45], induction of Type I IFN signaling via intratracheal Poly I:C administration still exacerbated TB in *Ch25h^−/−^* mice (Figure S9). These results indicated that Type I IFN‐mediated expression of CH25H does not significantly affect disease progression in the model of pulmonary Mtb infection. To further explore the differences in DC functions between BCG and Mtb challenges in WT and *Ch25h^−/−^* mice, we performed in vitro experiments using BMDCs. Notably, BCG exposure not only significantly enhanced IL‐12p70 production in *Ch25h^−/−^* BMDCs compared with WT, but also induced higher IL‐12p70 levels than Mtb infection, implying that CH25H predominantly affects DC‐mediated Th1 immunity following BCG vaccination rather than during Mtb infection (Figure S9). Particularly, distinct upregulation of IL‐2 from T cells activated by *Ch25h^−/−^* DCs provided us with novel clues about the role of CH25H deficiency in boosting anti‐TB immunity. Considering that IL‐2 plays a significant role in anti‐TB immunity, particularly by promoting Mtb‐specific CD4^+^ T cells activation and proliferation [46], we hypothesized that enhanced DC functions and DC‐mediated T cell activation resulting from CH25H deficiency might contribute to improving the BCG‐induced immunity, consistent with studies highlighting the role of IL‐2 in sustaining antigen‐specific immune responses [47, 48]. To confirm whether CH25H deficiency alters T cell responses induced by BCG vaccination, we subcutaneously vaccinated both WT and *Ch25h^−/−^* mice with BCG. Following BCG vaccination, the mice were euthanized at 10 weeks to assess immune functions (Figure 4A). BCG‐vaccinated *Ch25h^−/−^* mice exhibited an increased number of pulmonary CD44^hi^CD62L^lo^ effector T cells, characterized by higher expression of CD44 (Figure 4B). Ag85B‐specific CD4^+^ T cells and CD69^+^CD103^+^ TRM CD4^+^ T cells were increased in the lungs of BCG‐vaccinated *Ch25h^−/−^* mice (Figure 4C). Furthermore, effector T cells from BCG‐vaccinated *Ch25h^−/−^* mice exhibited increased Ki‐67 expression, a potential marker indicating heightened T cell proliferation [49]. They also featured decreased KLRG1 expression, which is indicative of a highly proliferative T cell phenotype [50] (Figure 4D). Pulmonary T cells of BCG‐vaccinated *Ch25h^−/−^* mice displayed significant differences in cytokine‐producing profiles in response to mycobacterial antigens PPD and Ag85B. The proportions of multifunctional IFN‐γ^+^TNF‐α^+^, IFN‐γ^+^IL‐2^+^, IFN‐γ^+^TNF‐α^+^IL‐2^+^ pulmonary CD4^+^ T cells (Figure 4E) and the numbers of IFN‐γ^+^, TNF‐α^+^, and IL‐2^+^ CD4^+^ T cells (Figure 4F) were increased in BCG‐vaccinated *Ch25h^−/−^* mice. Enhanced Mtb‐specific immune responses were reflected at the cytokine level, as the expression of IFN‐γ, TNF‐α, and IL‐2 was increased in lung cells as well. Notably, IL‐2 production was significantly upregulated in *Ch25h^−/−^* mice, suggesting its potential role in the rapid proliferation of antigen‐specific multifunctional T cells. However, the expression of IL‐10 was decreased in *Ch25h^−/−^* mice (Figure 4G). Although the number of pulmonary DCs and CD86 expression on DCs increased in BCG‐vaccinated *Ch25h^−/−^* mice, CD80 expression was not significantly altered (Figure S10A). Humoral responses against Mtb antigens were not strongly induced in BCG‐vaccinated *Ch25h^−/−^* mice, as the induction of PPD and Ag85B specific IgGs were not significantly increased (Figure S10B). Collectively, these findings suggest that CH25H restricts the optimal induction of anti‐TB immune memory in response to BCG vaccination.

**FIGURE 4:**
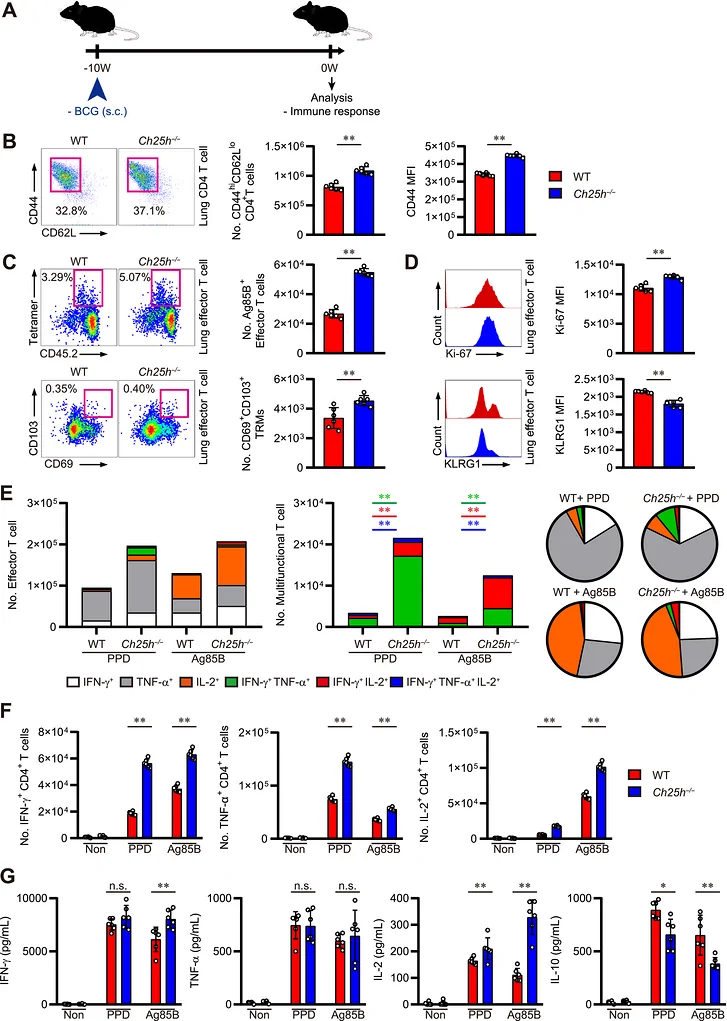
Immunological profiles of pulmonary CD4^+^ T cells in BCG‐vaccinated, uninfected WT and *Ch25h* ^−/−^ mice. (A) Experimental scheme for in vivo BCG vaccination. (B) Flow cytometry analysis of pulmonary effector CD4^+^ T cells. (C) Flow cytometry analysis of pulmonary Ag85B‐specific CD4^+^ T cells and CD69^+^CD103^+^ tissue‐resident memory T cells (TRMs). (D) MFI values of Ki‐67 and KLRG1 in pulmonary effector T cells. (E) The frequencies of Mtb‐specific pulmonary multifunctional T cells, represented as the percent of each cytokine positive T cell among the total cytokine positive T cells. Green, red, and blue bars indicate statistical significance between WT and *Ch25h* ^−/−^ mice for IFN‐γ^+^ TNF‐α^+^, IFN‐γ^+^ IL‐2^+^, and IFN‐γ^+^ TNF‐α^+^ IL‐2^+^ CD4^+^ T cells, respectively. (F) The numbers of Mtb‐specific IFN‐γ^+^, TNF‐α^+^, and IL‐2^+^ CD4^+^ T cells. (G) The levels of IFN‐γ, TNF‐α, IL‐2, IL‐17A, and IL‐10 in the supernatant of antigen stimulated lung cells in BCG‐vaccinated WT and *Ch25h* ^−/−^ mice. The data are presented as the mean ± SD of six mice in each group. The significance of differences was determined using the Mann–Whitney *U*‐test. *n.s*., not significant. *p < 0.05, **p < 0.01.

### 2.5 BCG‐Induced Mtb‐Specific Effector T Cells Provide Improved Protection Against Mtb in *Ch25h^−/−^* Mice

Next, we investigated whether CH25H deficiency enhances the protective immunity of BCG vaccination in vivo, and contributes to the long‐term maintenance of protection against Mtb. As clinical reports indicate that the efficacy of BCG diminishes during the later stages of Mtb infection [51, 52], BCG‐vaccinated WT and *Ch25h^−/−^* mice were infected with Mtb at 10 weeks postvaccination. We examined whether BCG‐vaccinated *Ch25h^−/−^* mice exhibit improved protection against Mtb at 6 weeks postinfection representing an early stage of infection (Figure 5A). While no discernible difference in TB progression was observed between unvaccinated WT and *Ch25h^−/−^* mice, BCG‐vaccinated *Ch25h^−/−^* mice showed a marked reduction in mycobacterial loads and pulmonary inflammatory lesions compared with WT mice (Figure 5B,C). The number of pulmonary effector T cells remained elevated in *Ch25h^−/−^* mice (Figure 5D). Intact Mtb‐specific T cell responses were represented by the induction of multifunctional IFN‐γ^+^TNF‐α^+^, IFN‐γ^+^IL‐2^+^, IFN‐γ^+^TNF‐α^+^IL‐2^+^ pulmonary CD4^+^ T cells (Figure 5E) and increased numbers of IFN‐γ^+^, TNF‐α^+^, and IL‐2^+^ CD4^+^ T cells (Figure 5F). Enhanced production of IFN‐γ, TNF‐α, and IL‐2, accompanied by reduced IL‐10 production, was observed (Figure 5G). Taken together, CH25H deficiency promotes the induction of Mtb‐specific Th1 response, leading to enhanced protection against Mtb in BCG‐vaccinated mice.

**FIGURE 5:**
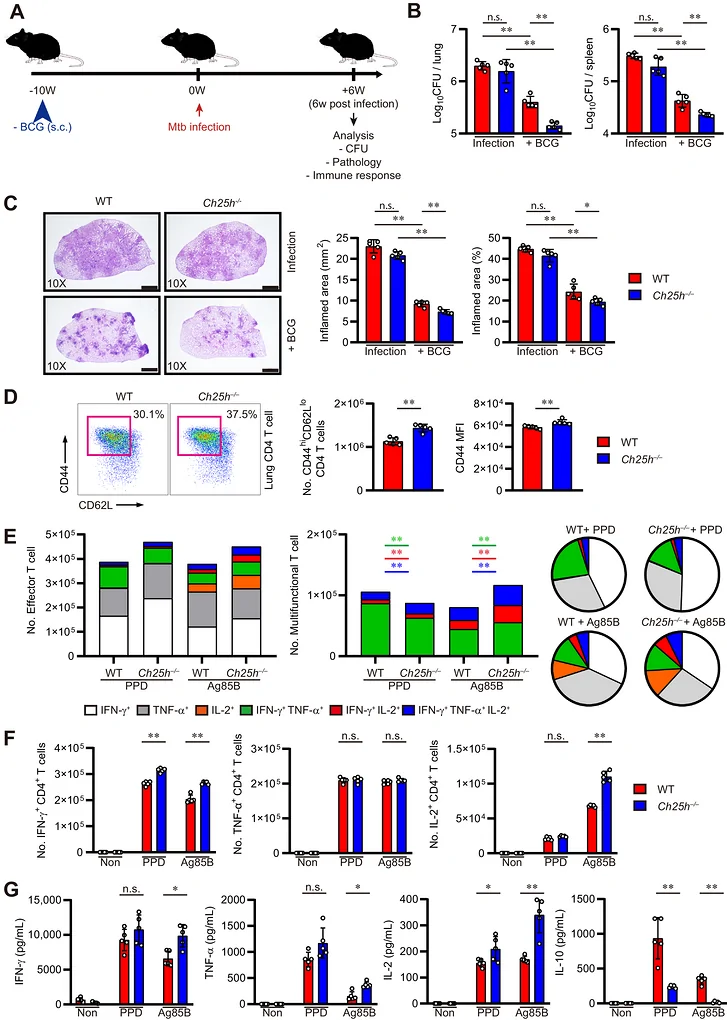
Analysis of TB progression and immunological profiles in WT and *Ch25h* ^−/−^ mice at 6 weeks postinfection following BCG vaccination. (A) Experimental scheme for in vivo Mtb challenge in BCG‐vaccinated mice. (B) CFUs of lungs and spleen and (C) pulmonary inflamed lesions of Mtb‐infected WT and *Ch25h* ^−/−^ mice at 6 weeks postinfection (scale bar = 2 mm). (D) Flow cytometry analysis of pulmonary effector CD4^+^ T cells. (E) The frequencies of Mtb‐specific pulmonary multifunctional T cells, represented as the percent of each cytokine positive T cell among the total cytokine positive T cells. Green, red, and blue bars indicate statistical significance between WT and *Ch25h* ^−/−^ mice for IFN‐γ^+^ TNF‐α^+^, IFN‐γ^+^ IL‐2^+^, and IFN‐γ^+^ TNF‐α^+^ IL‐2^+^ CD4^+^ T cells, respectively. (F) The numbers of Mtb‐specific IFN‐γ^+^, TNF‐α^+^, and IL‐2^+^ CD4^+^ T cells. (G) The levels of IFN‐γ, TNF‐α, IL‐2, IL‐17A, and IL‐10 in the supernatant of antigen stimulated Mtb‐infected lung cells in WT and *Ch25h* ^−/−^ mice. The data are presented as the mean ± SD of five mice in each group. The significance of differences was determined using the ANOVA test and Mann–Whitney *U*‐test. *n.s*., not significant. *p < 0.05, **p < 0.01.

### 2.6 CH25H Deficiency Promotes Long‐Term Maintenance of Mtb‐Specific Th1 Responses

To ascertain whether the improved protection against Mtb in BCG‐vaccinated *Ch25h^−/−^* mice can be sustained beyond 6 weeks postinfection, we evaluated the impact of CH25H deficiency on TB progression in *Ch25h^−/−^* mice at a later time point. Given that the efficacy of BCG vaccination is reported to diminish after 60 days in WT mice [53, 54], we conducted analysis at a time point beyond this threshold. At 12 weeks postinfection, the mice were euthanized to assess the severity of TB and immune functions (Figure 6A). Notably, BCG vaccinated *Ch25h^−/−^* mice exhibited a significant reduction in Mtb burden in lungs and spleen as well as pulmonary inflammation at 12 weeks postinfection (Figure 6B,C). Furthermore, we observed sustained Th1 responses against Mtb. Although the number of pulmonary effector T cells was not significantly altered (Figure 6D), multifunctional IFN‐γ^+^ TNF‐α^+^, IFN‐γ^+^IL‐2^+^, IFN‐γ^+^TNF‐α^+^IL‐2^+^ CD4^+^ T cells (Figure 6E) and IFN‐γ^+^, TNF‐α^+^, and IL‐2^+^ CD4^+^ T cells (Figure 6F) were strongly induced in the lungs of BCG‐vaccinated *Ch25h^−/−^* mice. In addition, although the expression of IFN‐γ and TNF‐α showed no difference, the expression of IL‐2 was significantly sustained, along with a reduction in IL‐10 expression (Figure 6G). Taken together, CH25H deficiency sustains and enhances BCG‐induced protective immunity by promoting durable Th1 responses.

**FIGURE 6:**
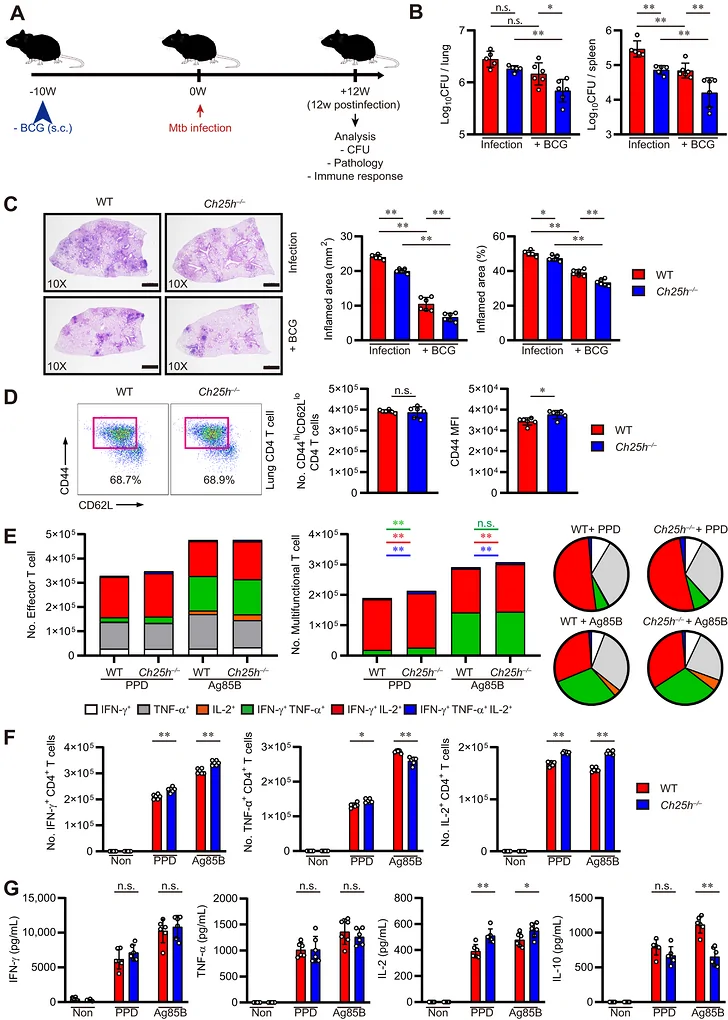
Ch25h deficiency sustained BCG‐induced pulmonary Th1 immunity and improved long‐term control of Mtb at 12 weeks postinfection. (A) Experimental scheme for in vivo Mtb challenge in BCG‐vaccinated mice. (B) CFUs of lungs and spleen and (C) pulmonary inflammatory lesions of Mtb‐infected WT and *Ch25h* ^−/−^ mice at 12 weeks postinfection (scale bar = 2 mm). (D) Flow cytometry analysis of pulmonary effector CD4^+^ T cells. (E) The frequencies of Mtb‐specific pulmonary multifunctional T cells, represented as the percent of each cytokine positive T cell among the total cytokine positive T cells. Green, red, and blue bars indicate statistical significance between WT and *Ch25h* ^−/−^ mice for IFN‐γ^+^ TNF‐α^+^, IFN‐γ^+^ IL‐2^+^, and IFN‐γ^+^ TNF‐α^+^ IL‐2^+^ CD4^+^ T cells, respectively. (F) The numbers of Mtb‐specific IFN‐γ^+^, TNF‐α^+^, and IL‐2^+^ CD4^+^ T cells. (G) The levels of IFN‐γ, TNF‐α, IL‐2, IL‐17A, and IL‐10 in the supernatant of antigen stimulated Mtb‐infected lung cells in WT and *Ch25h* ^−/−^ mice. The data are presented as the mean ± SD of six mice in each group. The significance of differences was determined using the ANOVA test and Mann–Whitney *U*‐test. *n.s*., not significant. *p < 0.05. **p < 0.01.

## 3 Discussion

To our knowledge, this study is the first to suggest CH25H deficiency enhances anti‐TB immunity through the DC‐T cell axis. We found that *Ch25h^−/−^* DCs exhibited increased CD80 and CD86 expression, elevated IL‐12p70 and reduced IL‐10, promoting T cell proliferation and Th1 cytokine secretion. *Ch25h^−/−^* mice showed enhanced Mtb‐specific effector T cells and lung TRMs following BCG vaccination. Moreover, BCG‐vaccinated *Ch25h^−/−^* mice developed highly proliferative Mtb‐specific T cells that persisted in the lungs for up to 12 weeks postinfection with Mtb, indicating improved mycobacterial clearance. Collectively, our findings suggest that CH25H acts as a negative regulator of DC‐driven Th1 immunity.

Notably, our data indicate that CH25H deficiency in DCs, rather than in T cells, is essential for the establishment of robust Mtb‐specific Th1 responses. Although CH25H is known to suppress inflammatory programs in innate immune cells [19, 32] and has been reported to promote T cell‐mediated inflammation in certain neuroinflammatory settings [23, 24], we observed no significant differences in Th1 cytokine production or proliferative capacity between WT and *Ch25h^−/−^* T cells following stimulation by antigen‐activated DCs. These findings suggest that CH25H deficiency in T cells does not critically influence immune responses to Mtb or BCG. Instead, our results identify DCs as the primary cellular compartment in which CH25H acts to constrain vaccine‐induced Th1 immunity, thereby limiting BCG‐mediated protective immunity.

Our transcriptomic analyses suggest that the CH25H/25‐HC axis may regulate *Il12a* expression through an SREBP2‐associated cholesterol biosynthetic program in BCG‐exposed DCs. Since 25‐HC is known to suppress SREBP signaling [55] and SREBP2‐related pathways have been implicated in innate inflammatory activation [56, 57] loss of CH25H‐derived 25‐HC may relieve suppression of the SREBP2 program, thereby contributing to enhanced *Il12a* expression and IL‐12p70 production. Additionally, because cholesterol and oxysterol metabolism regulate inflammation, we also investigated other pathways including LXR and SREBP1. LXR is an oxysterol‐responsive nuclear receptor that regulates cholesterol homeostasis and has been implicated in the modulation of inflammatory cytokine responses in antigen‐presenting cells, including IL‐12 family cytokines [58, 59]. Additionally, SREBP1 has been linked to innate immune activation by regulating lipid metabolic programs required for macrophage inflammatory responses, inflammasome, and TLR‐driven phagocytic activity [60, 61]. However, neither LXR‐ nor SREBP1‐related genes showed significant reciprocal alterations. Taken together, these findings suggest that CH25H deficiency‐mediated enhanced SREBP2 programming is associated with *Il12a* induction in BCG‐exposed *Ch25h* ^−/−^ DCs.

Although multifunctional Th1 responses are often associated with protection in preclinical TB vaccine studies [62, 63], they are not considered definitive correlates of vaccine efficacy in nonhuman primate models and clinical trials [64, 65, 66, 67]. Therefore, the enhanced multifunctional Th1 responses in our study represent promoted vaccine‐induced immune responses. Interestingly, we observed that BCG vaccination elicited highly proliferative effector T cells in the lungs of *Ch25h* ^−/−^ mice, a phenotype that may be associated with enhanced IL‐2 production, a central driver of T cell expansion, memory differentiation, and long‐term maintenance of antigen‐specific T cell responses [68, 69]. Previous studies have shown that IL‐2‐producing memory‐like CD4^+^ T cells contribute to sustained bacterial control during Mtb infection, and that maintaining memory CD4^+^ T cell responses in the lung is important for durable vaccine‐induced protection [70, 71]. Although the upstream mechanisms governing sustained IL‐2 induction in T cells remain incompletely defined beyond DC activation, we speculate that autocrine IL‐2 signaling within Th1 cells contributes to its sustained expression [72, 73]. Consistent with this notion, IL‐2‐producing T cells persisted in the lungs of BCG‐vaccinated *Ch25h* ^−/−^ mice for up to 12 weeks post‐Mtb infection, a stage at which antigen‐driven DC stimulation is likely diminished. In addition, the pro‐inflammatory milieu associated with *Ch25h* ^−/−^ innate cells may further support IL‐2 production, as cytokines such as IL‐1, IL‐15, and TNF‐α are known to promote IL‐2 expression in T cells [74]. Our data showed that exogenous 25‐HC treatment reduced T cell proliferation and decreased production of the relevant cytokine IL‐2, supporting the possibility that inhibition of 25‐HC may enhance IL‐2‐producing T cell responses. Together, our findings may provide an important clue for understanding the mechanisms that support the durability of vaccine‐induced protective immunity.

This study has several limitations. First, although our RNA‐seq and qPCR analyses suggest a potential link between the SREBP2‐associated program and *Il12a* induction, we did not directly examine SREBP2 activation or its causal role in *Il12a* regulation. Therefore, further approaches such as assessment of nuclear SREBP2 activation and pharmacological modulation of SREBP2 will be required to clearly define the mechanism. Second, we could not exclude the contribution of other immune and nonimmune cell populations to vaccine‐induced protective immunity in *Ch25h* ^−/−^ mice. Previous studies have shown that CH25H can modulate macrophage inflammatory cytokine production and inflammasome activity, limit IL‐17A‐producing T cells and neutrophilic inflammation, shape plasma cell differentiation, regulate epithelial barrier integrity, and influence endothelial ER stress during acute lung injury [20, 34, 75, 76]. Therefore, broader inflammatory alterations beyond DC‐intrinsic mechanisms also partially contribute to the BCG‐mediated protective immunity in *Ch25h* ^−/−^ mice. Last, our study was conducted using a subcutaneous BCG vaccination model; therefore, whether similar effects occur with other vaccination platforms, such as mucosal administration, remains unknown. Despite these limitations, our findings provide potential insight into how metabolic regulators can shape BCG‐induced protective immune responses.

Conventional approaches to TB vaccine development have primarily focused on generating new vaccine candidates or adjuvants that enhance immune responses against Mtb. In contrast, our study introduces a fundamental strategy that targets a detrimental host factor interfering with the induction of optimal anti‐TB immunity. These findings may also inspire further research to identify additional host factors that impair the optimal activation of pathogen‐specific immune memory elicited by vaccination.

In conclusion, CH25H constrains BCG‐induced immunity against Mtb. By suppressing DC activation and Th1 polarization, CH25H acts as an immune brake on the proliferation and maintenance of protective T cell responses. CH25H deficiency enhances Th1 priming and promotes sustained IL‐2‐producing T cell responses following BCG vaccination. These findings suggest that targeting CH25H may represent a potential strategy to improve BCG‐induced immune protection.

## 4 Materials and Methods

### 4.1 Mice

All mice were male and 6–7 weeks old. C57BL/6J and BALB/c mice were purchased from Japan SLC, Inc. (Shizuoka, Japan). P25 TCR‐Tg and *Ch25h^−/−^* mice were obtained from Jackson Laboratory (Sacramento, CA, USA). All animals were maintained under specific pathogen‐free conditions in a barrier facility at the Yonsei University Medical Research Center.

### 4.2 Bone Marrow‐Derived Cell Culture

Murine BMDCs were prepared and generated. Briefly, bone marrow cells were seeded onto petri dishes and maintained at 37°C in a humidified incubator containing 5% CO_2_. Cells were cultured in RPMI 1640 medium supplemented with 10% fetal bovine serum, 100 U/mL penicillin/streptomycin, 50 µM β‐mercaptoethanol, 20 ng/mL GM‐CSF, and 5 ng/mL IL‐4. Fresh medium was added on Day 3, and the culture medium was fully replaced on Day 6. On Day 8, all cells were harvested. Murine BMDMs were cultured as previously described [77].

### 4.3 Mycobacterial Culture and Antigens

*Mycobacterium bovis* BCG Pasteur 1137P2 was kindly gifted by Dr. Roland Brosch (Institut Pasteur, Paris, France). Mtb strain K was acquired from the Korean Institute of Tuberculosis (Osong, Republic of Korea). Mtb strain H37Rv was obtained from American Type Culture Collection (ATCC, Manassas, VA, USA). Mycobacteria were cultured in Middlebrook 7H9 broth supplemented with 10% oleic acid–albumin–dextrose–catalase (Difco Laboratories, NJ, USA) for 4 weeks at 37°C. Mycobacterial antigens PPD and Ag85B were used as stimuli, and LPS (from *E. coli* O111:B4) purchased from InvivoGen (San Diego, CA, USA) served as a control.

### 4.4 mRNA Sequencing

Total RNA was isolated from BMDCs, and RNA quality was assessed using the TapeStation 4000 system (Agilent Technologies, Santa Clara, CA, USA). Poly(A)‐enriched RNA libraries were prepared using the CORALL RNA‐Seq V2 Library Prep Kit (Lexogen GmbH, Vienna, Austria) and sequenced on an Illumina platform (Illumina, San Diego, CA, USA) with paired‐end reads. Sequencing reads were quality‐filtered, aligned to the mouse reference genome, and quantified using fastp, STAR, and Salmon. Gene expression data were normalized using CPM/TMM normalization, and differentially expressed genes were identified using ExDEGA software (Ebiogen, Seoul, Republic of Korea). To account for basal genotype differences, BCG‐induced transcriptional changes were analyzed relative to genotype‐matched naive controls.

### 4.5 BMDC Activation and Quantification of T Cell Responses via DC‐T Cell Coculture

Splenocytes from C57BL/6J, *Ch25h^−/−^*, and BALB/c mice were used for allogeneic DC‐T cell cocultures. For the assessment of Mtb antigen‐specific T cell proliferation and activation, splenocytes were obtained from P25 TCR‐Tg mice and from BCG‐immunized C57BL/6J mice at 10 weeks postvaccination. CD90.2^+^ T cells were isolated from spleen cell suspensions using a MACS column. Splenic T cells were stained with 1 µM CFSE (Invitrogen, Waltham, MA, USA) or CellTrace Violet (CTV) (Invitrogen). Subsequently, CFSE or CTV‐labeled T cells were cultured with stimulated BMDCs. The DCs were stimulated with PPD (1 µg/mL) or Ag85B peptide (5 µg/mL) and seeded at 2 × 10^5^ cells per well at a DC: T cell ratio of 1:6. After 3 days of coculture, the T cells were stained with monoclonal antibodies against CD90.2, CD4, CD8, and CD44 and the cell proliferation rate was analyzed using a CytoFLEX S Flow cytometer (Beckman Coulter, Indianapolis, IN). Supernatants were harvested, and cytokine production from splenic T cells was quantified using mouse IFN‐γ, TNF‐α, IL‐2, and IL‐17A ELISA kits (Invitrogen) as well as a mouse IL‐10 ELISA kit (BioLegend, San Diego, CA, USA).

### 4.6 BCG Vaccination and Mtb Infection

Mice were vaccinated with BCG via subcutaneous injection (1.0 × 10^6^ colony forming units (CFUs)/mouse). Ten weeks after vaccination, the vaccinated mice were autopsied for evaluating immunological features. For Mtb infection, aerosol challenge was performed by a Glas‐Col aerosol apparatus (Glas‐Col, Terre Haute, IN, USA) for 70 min, adjusted to achieve an initial infectious dose of 200 CFUs. At 4, 6, and 12 weeks postinfection, mice from each group were euthanized for analysis of the Mtb loads, histopathology, and immunological profiles.

### 4.7 Preparation of Single Cell Suspensions From Mice

The mice undergoing in vivo analysis of immune responses and TB progression were euthanized via inhalation of carbon dioxide. Lungs and spleens of autopsied mice were obtained to assess bacterial load, inflammation, and immune cell distribution. According to the methods illustrated in previous studies [78], single‐cell suspensions were harvested from the lung tissue and went through flow cytometry analysis or coculture with DCs.

### 4.8 Enumeration of Mycobacterial CFU and Assessment of Pulmonary Inflammation

The lungs and spleens of Mtb‐infected mice were harvested after autopsy. To determine CFU, lung and spleen tissues were plated onto Middlebrook 7H10 agar (BD, Franklin Lakes, NJ, USA) after homogenization, following preparation methods from previous studies. After a 4‐week incubation period at 37°C, mycobacterial colonies were counted. To enumerate lung inflammation, the right superior lobes of the lungs were preserved in 10% formalin overnight and later embedded in paraffin. To perform histopathologic analysis, the lungs were sectioned at 4–5 µm and stained with hematoxylin and eosin. The pulmonary inflamed lesions were quantified by the ImageJ software.

### 4.9 Antibodies and Flow Cytometry

Flow cytometry analysis of immune cells was conducted using the following antibodies. LIVE/DEAD Stain Kit was purchased from Molecular Probes (Carlsbad, CA, USA). BV605‐conjugated mAb against Thy1.2 and CD19; APC‐conjugated mAb against CD45R; BV421‐conjugated mAb against CD45; APC‐R700‐conjugated mAb against Siglec‐F; FITC‐conjugated mAb against CD45.2; Alexa Fluor 488‐conjugated mAb against IL‐17A; and V450‐conjugated mAb against Ly6G were purchased from BD. BV421‐conjugated mAb against CD86, MHC‐II, KLRG1, CD44, and CD62L; BV785‐conjugated mAb against CD8; PE‐conjugated mAb against CD80, CD64, and IFN‐γ, and PerCP‐Cy5.5‐conjugated mAb against CD4 and CD11b; APC‐conjugated mAb against TNF‐α, Ki‐67, and CD103; PE‐Cy7‐conjugated mAb against PD‐1; APC‐Cy7‐conjugated mAb against MHC‐II; PE‐Cy7‐conjugated mAb against IL‐2; Alexa Fluor 700‐conjugated mAb against CD62L; and PE‐Dazzle conjugated mAb against CD11c and CD69 were purchased from BioLegend. PE‐conjugated anti Ag85B specific tetramers were kindly obtained from the NIH Tetramer Core Facility (Emory University, Atlanta, GA, USA).

### 4.10 Ex Vivo Analysis of Multifunctional T Cell Responses

Mouse lung cells and splenocytes containing T cells were stimulated with PPD (1 µg/mL) or Ag85B (5 µg/mL) for 12 h at 37°C. Following stimulation, supernatants and cell pellets were harvested for ELISA and flow cytometry analysis. Cell pellets underwent Fc‐receptor blockade using anti‐CD16/32 antibody (BioLegend) before surface staining. After surface staining, cells were permeabilized with BD Perm/Fix solution (BD) and intracellularly stained. Cells were fixed with IC fixation buffer (Invitrogen) and analyzed using a CytoFLEX S Flow cytometer (Beckman Coulter, Brea, CA, USA). The analysis of cytokine secretion from the supernatants was performed using the mouse ELISA kits listed below.

### 4.11 Real‐Time Quantitative PCR

Primers and probes for CH25H were designed based on nucleotide sequences from our previously cloned gene or retrieved from Kyoto Encyclopedia of Genes and Genomes (Kyoto, Japan) and Bionics (Seoul, Republic of Korea) synthesized the primers. The primer sequences used for qPCR were as follows: *Il12a* forward, 5′‐GTG CCT TGG TAG CAT CTA TGA‐3′ and reverse, 5′‐TGC CCT TGT CTA GAA TGA TCT G‐3′; *Il12b* forward, 5′‐AGC ACG GCA GCA GAA TAA A‐3′ and reverse, 5′‐CTC CAC CTG TGA GTT CTT CAA A‐3′; *Ch25h* forward, 5′‐CTC ACA GAC TTG TGC CCT TT‐3′ and reverse, 5′‐TTG TCC CAG TGT GTG AAG TAA G‐3′; *Hmgcr* forward, 5′‐GAC GAA GAT GTG TCC AAG AGA A‐3′ and reverse, 5′‐GGA AGG CTA AAC TCA GGG TAA TC‐3′; *Hmgcs1* forward, 5′‐CGA TGG TGT AGA TGC TGG AAA‐3′ and reverse, 5′‐GAA CCA CAG TCA GGC AAA GA‐3′; *Fdps* forward, 5′‐TCG GGT GAA AGC ACT GTA TG‐3′ and reverse, 5′‐GCA CTG CTC TAT GAG ACT CTT G‐3′; *Sqle* forward, 5′‐AGA GCC CGA CAG GAT AGT T‐3′ and reverse, 5′‐GAT GGG CAT TGA GAC CTT CTA C‐3′; and *Actb* forward, 5′‐CCG TAA AGA CCT CTA TGC CAA C‐3′ and reverse, 5′‐AGG AGC CAG AGC AGT AAT CT‐3′. Real‐time quantitative PCR (RT‐qPCR) was performed using a StepOnePlus Real‐Time PCR System (Thermo Fisher Scientific, Waltham, MA, USA). The 10 µL reaction volume contained 5 µL of TB Green Premix Ex Taq 2× SYBR Green (Takara Bio Inc., Shiga, Japan), 1 µL each of forward and reverse primers, 1 µL of cDNA template, and 2 µL of nuclease‐free water. Thermal cycling conditions included initial denaturation at 95°C for 30 s, followed by 40 cycles of denaturation at 95°C for 5 s and annealing/extension at 62°C for 30 s. Gene expression levels were quantified using the 2^(−ΔΔCt)^ method, with β‐actin as the internal reference gene. All samples were analyzed in triplicate.

### 4.12 Measurement of Cytokines and IgG Responses

Lung‐derived single‐cell suspensions were restimulated with mycobacterial antigens, PPD or Ag85B. Cytokine levels were quantified using mouse IFN‐γ, TNF‐α, IL‐1β, IL‐2, IL‐12p70, and IL‐17A ELISA kits purchased from Invitrogen. A Mouse IL‐10 ELISA kit was purchased from BioLegend. For the analysis of serum IgGs, biotin‐conjugated antimouse total IgG antibody was purchased from Sigma–Aldrich (Burlington, MA, USA). Biotin‐conjugated antimouse IgG1 and IgG2c antibodies were obtained from Biowest (Bradenton, FL, USA). Antibiotin HRP and substrate solution were purchased from Invitrogen.

### 4.13 Statistical Analyses

The results were presented as the mean ± standard deviation (SD). For comparisons between the two groups, an unpaired *t*‐test or Mann–Whitney *U*‐test was conducted. To analyze the significance of differences between more than two selected groups, an ANOVA test was conducted. GraphPad Prism version 8.0 (GraphPad Software, La Jolla, CA, USA) was used for statistical analysis. The statistical significance was determined using the following definitions: *n.s*.: not significant, *p < 0.05, **p < 0.01, ***p < 0.001, ****p < 0.0001.

## Author Contributions

Sung Jae Shin conceived and supervised the study. Hagyu Kim, Eunsol Choi, Kee Woong Kwon, Kyungmin Kim, and WeonSeok Jung performed the investigation, and Hagyu Kim, Eunsol Choi, and Hong‐Hee Choi designed and implemented the methodology. Hagyu Kim and Eunsol Choi drafted the original manuscript, and Hagyu Kim, Eunsol Choi, and Sung Jae Shin reviewed and revised the manuscript. All authors approved the final version of the manuscript.

## Funding

This work was supported by the National Research Foundation of Korea (NRF) grant funded by the Ministry of Science and ICT (MSIT) (RS‐2023‐00208115), the Korea Health Technology R&D Project through the Korea Health Industry Development Institute (KHIDI) funded by the Ministry of Health & Welfare, Republic of Korea (RS‐2025‐02173063), and a grant from the Ministry of Food and Drug Safety (26202MFDS013) in 2026.

## Ethics Statements

All in vivo experiments were conducted in strict accordance with the guidelines established by the Korean Food and Drug Administration. Experimental procedures involving animals were approved by the Ethics Committee and the Institutional Animal Care and Use Committee of Yonsei University Health System (Seoul, South Korea) under the following each experimental number (2018‐0196; C57BL/6J, BALB/c, *Ch25h^−/−^*, 2021‐0159; C57BL/6J, BALB/c, *Ch25h^−/−^*, P25 TCR‐Tg).

## Conflicts of Interest

The authors declare no conflicts of interest.

## Supporting information




**Supporting File 1**: mco270989‐sup‐0001‐SuppMat.docx

## Data Availability

Data are available from the corresponding authors upon reasonable request. RNA sequencing data are available in the NCBI Gene Expression Omnibus (GEO) with accession code GSE 335794.
