# Supplementary material for: Cholesterol 25‐Hydroxylase Deficiency Provides an Optimal and Durable Protective T Helper 1 Immunity via Interleukin‐12–Interleukin‐2 Axis in BCG‐Vaccinated Mice
Source: MedComm (2020). 2026 Sep 30;7(10):e70989. doi: 10.1002/mco2.70989 (PMC13625813; doi:10.1002/mco2.70989)
Supplement: Supplementary file 1 [file MCO2-7-e70989-s001.docx]

**Cholesterol 25-hydroxylase deficiency provides an optimal and durable protective T helper 1 immunity via interleukin-12-interleukin-2 axis in BCG-vaccinated mice**

Running title: Role of CH25H in BCG-induced protective immunity

Hagyu Kim^1,2#^, Eunsol Choi^1#^, Kee Woong Kwon^1,3^, Hong-Hee Choi^1^, Kyungmin Kim^4^, WeonSeok Jung^1^, Sung Jae Shin^1, 5*^

^#^ These authors contributed equally to the work.

^1^Department of Microbiology, Institute for Immunology and Immunological Disease, Graduate School of Medical Science, Brain Korea 21 Project, Yonsei University College of Medicine, Seoul, South Korea

^2^Department of Microbiology, Yonsei University College of Medicine, Seoul 03722, South Korea

^3^Department of Microbiology, College of Medicine, Gyeongsang National University, Jinju, South Korea

^4^Department of Microbiology, Graduate School of Medicine, Yonsei University College of Medicine, Seoul, South Korea

^5^Department of Biomedical Sciences, Yonsei University College of Medicine, Seoul, Republic of Korea.

*Address correspondence to Sung Jae Shin, sjshin@yuhs.ac

Figure S1 Gating strategies for DCs and additional properties of *Ch25h^-/-^* BMDCs. (A) *In vitro* flow cytometry gating strategy for BMDCs. (B) Relative MFI values (% of unstimulated controls) of MHC-I and MHC-II in BMDCs after stimulation. (C) The expression levels of TNF-α, IL-6, IL-23 and TGF-β in the supernatants of stimulated BMDCs. (B-C) Data are presented as the mean ± SD of five biological replicates per group. The significance of differences was determined using an unpaired *t*-test. *n.s.*, not significant, **p* <0.05, ***p* <0.01, *****p* <0.0001 n.d., not detected

Figure S2 Comparison of cytokine production in *Ch25h^-/-^* and WT BMDMs following stimulation. BMDMs from WT and *Ch25h^-/-^* were exposed to antigen or BCG, or infected with Mtb for 24 h. The expression levels of IL-1β, IL-10, and IL-12p70 in the supernatants of (A) LPS- and PPD-stimulated, (B) BCG-exposed, and Mtb-infected BMDMs. Data are presented as the mean ± SD of four or five biological replicates per group. The significance of differences was determined using an unpaired *t*-test. *n.s.*, not significant, ***p* <0.01, n.d., not detected

Figure S3 RNA sequencing analysis of WT and *Ch25h*^-/-^ BMDCs following BCG exposure and 25-HC treatment. (A) Schematic overview of the mRNA sequencing analysis. (B) Shared genes showing reciprocal regulation in comparative heat map analysis. (C) Reactome pathway analysis of the shared genes. (D) GSEA analysis of cholesterol homeostasis. Heat maps of SREBP2 pathway-associated genes in (E) WT and *Ch25h*^-/-^ BMDCs and (F) *Ch25h*^-/-^ BMDCs with or without 10 μM 25-HC treatment. Validation of gene expression of (G) *Il12a* and *Il12b* and (H) SREBP2 pathway-associated genes in WT and *Ch25h*^-/-^ BMDCs. Validation of gene expression of (I) *Il12a* and *Il12b* and (J) SREBP2 pathway-associated genes in *Ch25h*^-/-^ BMDCs with or without 25-HC treatment. (G-J) Data are presented as the mean ± SD of three biological replicates per group. The significance of differences was determined using an unpaired *t*-test. *n.s.*, not significant, **p* <0.05, ***p* <0.01, ****p* <0.001, *****p* <0.0001

Figure S4 Gating strategies for T cells. (A) Flow cytometry gating strategy for T cells co-cultured with BMDCs. (B) Flow cytometry gating strategy for pulmonary cytokine producing T cells *in vivo*.

Figure S5 T cell proliferation and cytokine production of splenic T cells co-cultured with unstimulated WT and *Ch25h^-/-^* DCs. (A) Schematic overview of the unstimulated DC–T cell co-culture assay. Unstimulated WT and *Ch25h^-/-^* BMDCs were co-cultured with CFSE-labeled BALB/c splenic T cells. After three days of co-culture, T cell proliferation and cytokine production were analyzed by flow cytometry and ELISA, respectively. (B) Proliferation profiles of CD4⁺ and CD8⁺ BALB/c T cells co-cultured with WT or *Ch25h^-/-^* BMDCs. (C) The expression levels of IFN-γ, TNF-α, IL-2, IL-17A, and IL-10 in supernatants of BALB/c splenic T cells incubated with WT BMDC or *Ch25h^-/-^* BMDCs. (D) Proliferation of CD4^+^ and CD8^+^ T cell from WT or *Ch25h^-/-^* mice co-cultured with BALB/c BMDCs. (E) The expression levels of IFN-γ, TNF-α, IL-2, IL-17A, and IL-10 in supernatants of BALB/c BMDCs incubated with WT splenic T cells or *Ch25h^-/-^* splenic T cells. The data are presented as the mean ± SD of five biological replicates per group. The significance of differences was determined using unpaired *t*-test. *n.s.*, not significant. **p* <0.05. ***p* <0.01, ****p* < 0.001

Figure S6 Analysis of T cell proliferation and cytokine production in splenic T cells co-cultured with BCG-exposed WT and *Ch25h^-/-^* BMDCs. (A) Experimental scheme for DC-T cell co-culture assay. WT and *Ch25h^-/-^* BMDCs were exposed with BCG and co-cultured with CFSE-stained splenic T cells for three days. (B) CD4^+^ and CD8^+^ T cell proliferation status in BALB/c splenic T cells incubated with WT or *Ch25h^-/-^* BMDCs. (C) The expression levels of IFN-γ, TNF-α, IL-2, IL-17A, and IL-10 in supernatants of BALB/c splenic T cells incubated with WT BMDC or *Ch25h^-/-^* BMDCs. (D) Assessment of CD4^+^ and CD8^+^ T cell proliferation in WT or *Ch25h^-/-^* splenic T cells co-cultured with BALB/c BMDCs. (E) The expression levels of IFN-γ, TNF-α, IL-2, IL-17A, and IL-10 in supernatants of BALB/c BMDCs co-culture with WT splenic T cells or *Ch25h^-/-^* splenic T cells. Data are presented as the mean ± SD of five biological replicates per group. The significance of differences was determined using unpaired *t*-test. *n.s.*, not significant. **p* <0.05, ***p* <0.01, ****p* < 0.001, *****p* < 0.0001

Figure S7 DC-T cell co-culture assay with 25-HC treatment in WT and *Ch25h^-/-^* BMDCs. (A) Representative flow cytometry plots and quantification of CD44⁺ CellTrace Violet (CTV)^low^ CD4⁺ T cells after co-culture with WT or *Ch25h*^-/-^ DCs in the presence of 1 μM or 10 μM 25-HC. (B) IFN-γ, IL-2, and IL-10 levels in culture supernatants measured by ELISA. Data are presented as the mean ± SD of four biological replicates per group. Statistical significance was determined by ANOVA. **p* <0.05, ***p* <0.01, *****p* < 0.0001

Figure S8 Evaluation of TB progression and immune responses in WT and *Ch25h^-/-^* mice at four weeks post-infection. (A) Experimental scheme for *in vivo* Mtb challenge. (B) CFUs of lungs and spleen and (C) lung pathology of Mtb-infected WT and *Ch25h^-/-^* mice (scale bar = 2 mm). (D) Flow cytometry analysis of pulmonary DCs in Mtb-infected mice at four weeks post-infection. (E) Flow cytometry analysis of pulmonary effector CD4^+^ T cells. (F) The frequencies of Mtb-specific pulmonary multifunctional T cells, represented by the percent of each cytokine positive T cell among the total cytokine positive T cells. Green, red, and blue bars indicate statistical significance between WT and *Ch25h^-/-^* mice for IFN-γ^+^ TNF-α^+^, IFN-γ^+^ IL-2^+^, and IFN-γ^+^ TNF-α^+^ IL-2^+^ CD4^+^ T cells, respectively. (F) The numbers of Mtb-specific IFN-γ^+^, TNF-α^+^, and IL-2^+^ CD4^+^ T cells. (G) The numbers of Mtb-specific IFN-γ^+^, TNF-α^+^, and IL-2^+^ CD4^+^ T cells. (H) The expression levels of IFN-γ, TNF-α, IL-2, IL-17A, and IL-10 in the supernatants of antigen stimulated Mtb-infected lung cells from WT and *Ch25h^-/-^* mice. The data are presented as the mean ± SD of five mice in each group. The significance of differences was determined using the Mann-Whitney U test. *n.s.*, not significant. ***p* <0.01

Figure S9 Assessment of TB pathogenesis in WT and *Ch25h^-/-^* mice with Poly I:C administration. (A) Experimental scheme for *in vivo* Poly I:C treatment in Mtb-infected mice. Starting at 14 days post-infection, 200 µg of Poly I:C was administered intratracheally to the mice. The injections were performed once daily from 14 to 16 days post-infection. (B) CFUs of lungs and spleen and (C) lung pathology of Mtb-infected WT and *Ch25h^-/-^* mice (scale bar = 2 mm). H&E staining was performed at four weeks post-infection. The data are presented as the mean ± SD of five mice in each group. The significance of differences was determined using the ANOVA test. *n.s.*, not significant, **p* <0.05, ***p* <0.01

Figure S10 Level of IL-12p70 and IL-10 production in BCG-exposed or Mtb-infected WT and *Ch25h^-/-^* BMDCs. Data are presented as the mean ± SD of four biological replicates per group. The data were analyzed by one-way ANOVA with post hoc Tukey’s test. *n.s.*, not significant. *****p* <0.0001

Figure S11 Analysis of CD80 and CD86 expression of DC and IgG responses in BCG-vaccinated WT and *Ch25h^-/-^* mice. (A) Flow cytometry analysis of pulmonary DCs. (B) Evaluation of IgG, IgG1, and IgG2c levels in BCG-vaccinated mice. The data are presented as the mean ± SD of six mice in each group. The significance of differences was determined using Mann-Whitney *U* test. *n.s.*, not significant, **p* <0.05, ***p* <0.01
